# Early Spring

**Published:** 1871-04

**Authors:** 


### EARLY SPRING.

In March and early April the fitful weather is often more trying to the constitution than the steady and severe cold of midwinter, resulting in the death of many from pneumonia, that is, inflammation of the lungs. Such results are forwarded sometimes by changing the warm winter clothing for a lighter kind. Many lives might be saved every year, if no winter garment was laid aside before May. It is always safer to be dressed too warmly than to have so little clothing as to allow the body to become chilly. The rule should be to dress abundantly warm, at least until the spring weather has become more settled.

Colds may often be avoided, if, when there is a feeling of even slight chilliness, a brisk walk were taken and continued until a pleasant glow is felt all over the body; for the want of this, or its equivalent in some form of exercise or work, the chilliness has continued and increased until a cold has been taken which it may require weeks to cure.

If persons can manage to keep the feet and hands naturally and comfortably warm, such a thing as a bad cold would seldom be taken. It is through cold feet and cold hands that troublesome diseases come to multitudes.

